# The microRNA miR-33a suppresses IL-6-induced tumor progression by binding Twist in gallbladder cancer

**DOI:** 10.18632/oncotarget.12693

**Published:** 2016-10-15

**Authors:** Mingdi Zhang, Wei Gong, Bin Zuo, Bingfeng Chu, Zhaohui Tang, Yong Zhang, Yong Yang, Di Zhou, Mingzhe Weng, Yiyu Qin, Mingzhe Ma, Alex Jiang, Fei Ma, Zhiwei Quan

**Affiliations:** ^1^ Department of General Surgery, Xinhua Hospital, Shanghai Jiaotong University, School of Medicine, Shanghai, 200092, China; ^2^ Department of Anesthesiology and Surgical Intensive Care Unit, Xinhua Hospital, Shanghai Jiaotong University School of Medicine, Shanghai, 200092, China; ^3^ Department of Orthopedic Surgery, Xinhua Hospital, Shanghai Jiaotong University, School of Medicine, Shanghai, 200092, China; ^4^ Schulich School of Medicine and Dentistry, Western Ontario University, London, ON N6A 3K6, Canada; ^5^ Department of Oncology, Xinhua Hospital, Shanghai Jiaotong University, School of Medicine, Shanghai, 200092, China

**Keywords:** gallbladder cancer, microRNAs, epithelial–mesenchymal transition, twist, interleukin-6

## Abstract

Cytokine is a key molecular link between chronic inflammation and gallbladder cancer (GBC) progression. The potential mechanism of cytokine-associated modulation of microRNAs (miRNAs) expression in GBC progression is not fully understood. In this study, we investigated the biological effects and prognostic significance of interleukin-6 (IL-6) -induced miRNAs in the development of GBC. We identify that inflammatory cytokine, IL-6 promotes proliferation, migration, invasion and epithelial-mesenchymal transition (EMT) of GBC both *in vitro* and *in vivo*. Among all the changed miRNAs in miRNA profiling, miR-33a expression was significantly decreased in IL-6 treated GBC cell lines, as well as in GBC tissues compared with case-matched normal tissues and cholecystitis tissues. In turn, miR-33a suppresses IL-6−induced tumor metastasis by directly binding Twist which was identified as an EMT marker. High expression of miR-33a suppressed xenograft tumor growth and dissemination in nude mice. The downregulation of miR-33a was closely associated with advanced clinical stage, lymph node metastasis, and poor clinical outcomes in patients with GBC. miR-33a acts as a tumor suppressor miRNA in GBC progression and may be considered for the development of potential therapeutics against GBC.

## INTRODUCTION

Gallbladder cancer (GBC) is a rare neoplasm with an incidence of 2.5 in 100 000 individuals [1, 2]. Complete surgical resection is the only effective treatment, however, even after complete surgical resection, loco-regional recurrence rates are extremely high [3, 4]. The overall 5-year survival rate for GBC is less than 5% [5]. Local tumor growth, hepatic invasion, and lymph node metastasis are the main prognostic factors in patients with GBC [6].

The tumor-associated inflammatory response had the unanticipated, paradoxical effect of enhancing tumorigenesis and progression. Gallstones which may leads to chronic inflammation due to mechanical obstruction are found to be presented in about 65–90% of GBC patients [7]. The relationship between cytokines and progression of GBC has long been suspected [8, 9]. Among the cytokines reported so far, interleukin-6 (IL-6) is one of the pivotal proinflammatory cytokines presented in the tumor microenvironment [10–12]. However, the potential roles of IL-6 in the progression of GBC are still unclear.

Post-transcriptional regulation of gene expression activated by microRNAs (miRNAs) correlates with tumorigenesis and cancer metastasis [13–15]. Their emerging roles in the development and progression of human cancers not only provide prognostic information on tumor development, but also may represent novel diagnostic and therapeutic opportunities [4]. The functional relevance of miRNAs in GBC progression has been poorly documented [16–18].

In this study, we first identified miR-33a as a commonly downregulated miRNA in IL-6 treated GBC cells by using comparative miRNA profiling. Decreased miR-33a is associated with poor prognosis in GBC samples, which in turn promotes the metastasis of GBC both *in vitro* and *in vivo* by directly binding Twist. Moreover, we found that the downregulation of miR-33a in response to IL-6 treatment is necessary for IL-6 induced cell proliferation, migration and invasion, implying that miR-33a is inhibited in the IL-6-induced progression of GBC, and uncover a potential prognostic biomarker and molecular target for the treatment of GBC.

## RESULTS

### IL-6 promotes the proliferation and metastasis of GBC *in vitro*

**Figure 1:**
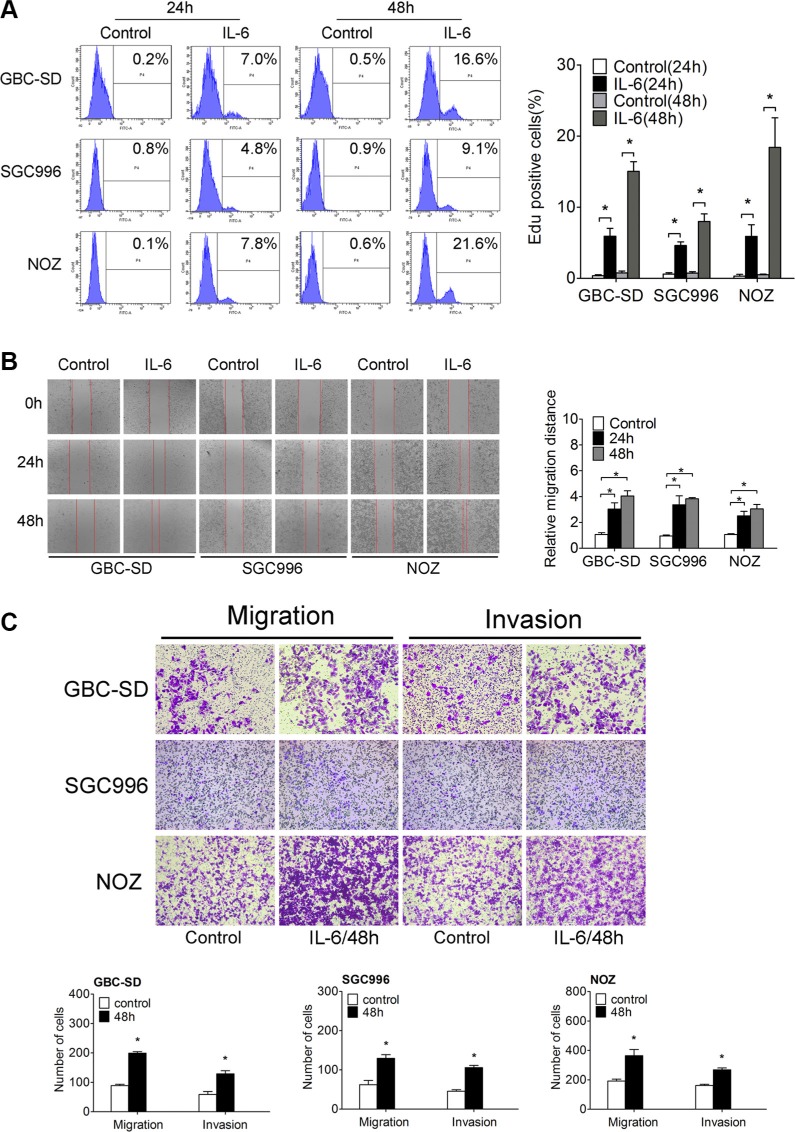
IL-6 promotes proliferation, migration and invasion of GBC *in vitro* (**A**) Flow cytometry analyzed the Edu positive cells after IL-6 treatment (Left panel). Quantitative analysis of percentage of Edu positive cells (Right panel), *P < 0.05. The results are shown as mean ± S.D. (**B**) The wound-healing assay of GBC cells after IL-6 treatment (Left panel). Quantitative analysis of migration distance (Right panel), *P < 0.05. The results are shown as mean ± S.D. (**C**) The transwell migration and invasion assay of GBC cells after IL-6 treatment (upper panel). Quantitative analysis of migrated cells (lower three panels), *P < 0.05. The results are shown as mean ± S.D.

To investigate the role of IL-6 in GBC cells, we first treated three GBC cell lines (GBC-SD, SGC-996, NOZ) continuously with several different concentrations of IL-6 (1 ng/ml, 10 ng/ml, 100 ng/ml, 200 ng/ml, 300 ng/ml, 400 ng/ml) for 72 h. Cell counting kit-8 (CCK-8) assay indicated that GBC cell proliferation was significantly promoted by IL-6 at lower concentrations (< 100 ng/ml) but was then gradually decreased at the higher concentrations over 100 ng/ml (Supplementary Figure S1A). We then used 100 ng/ml as the cutoff concentration for further investigation. Additionally, our results also showed that the percent of live GBC cells was significantly increased by IL-6 (Supplementary Figure S1A). Correspondingly, flow cytometry and immunofluorescence (IF) staining showed that the number of both BrdU- and EdU-positive cells was obviously greater in IL-6 treated cells in comparison with control cells (Supplementary Figure S1B and Figure 1A). Furthermore, colony-formation assay showed that IL-6 increased the colony number, area of single clone and clone diameter in three GBC cell lines (Supplementary Figure S1C). Thus, all these results confirm that IL-6 promotes GBC cell proliferation.

To explore the function of IL-6 on GBC migration and invasion, we treated the three GBC cell lines with IL-6. Wound healing and transwell migration assays demonstrated that migration/invasion was significantly increased by IL-6 treatment in GBC cells (Figure 1B and Figure 1C).

CD44^+^CD133^+^ cells which represent cancer stem cells (CSCs) in GBC possess the potentials for self-renewal and differentiation, tumorigenicity, extensive proliferation and high recurrence of tumors [19]. We then investigated the CD44^+^CD133^+^ population in IL-6 treatment GBC cells. Flow cytometry analysis demonstrated much higher CD44^+^CD133^+^ fractions in IL-6-treated GBC cells compared with control group (Supplementary Figure S1D). Overall, our results indicate that IL-6 can exert significant promotive effect on GBC cell migration and invasion *in vitro*.

### IL-6 promotes the proliferation and metastasis of GBC *in vivo*

**Figure 2:**
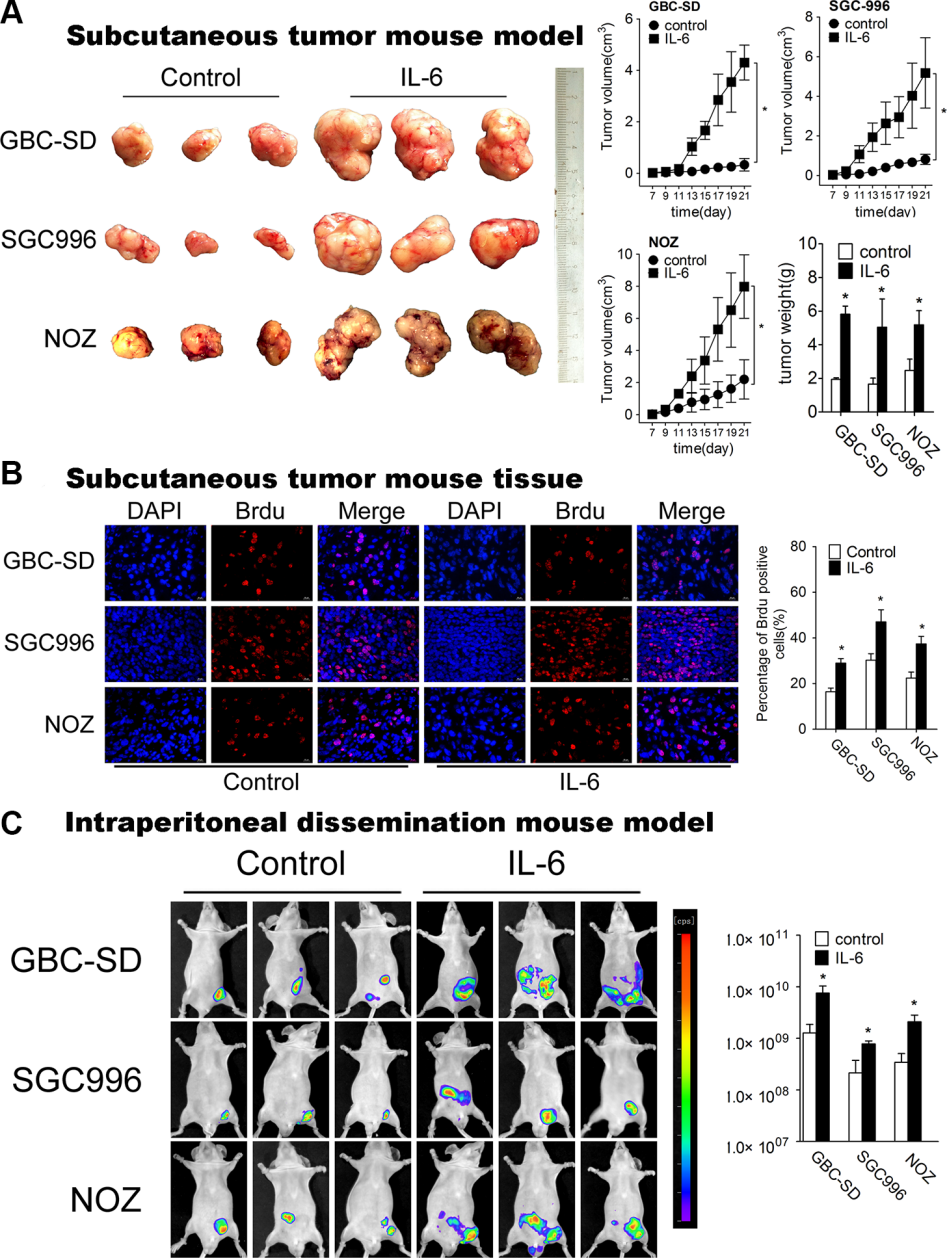
IL-6 promotes proliferation, metastasis of GBC *in vivo* (**A**) Mice bearing GBC-SD, SGC996 and NOZ subcutaneous tumor models after IL-6 treatment (*n* = 6 for each group). The right panel shows the volume and weight of the tumors, *P < 0.05. The results are shown as mean ± S.D. (**B**) Brdu positive cells measured by immunofluorescence in subcutaneous tumor tissues (×200) (Left panel). Quantitative analysis of percentage of Brdu positive cells (Right panel), *P < 0.05. The results are shown as mean ± S.D. (**C**) Nude mice engrafted with intraperitoneal GBC-luc tumors at 48 days after cell engraftment (left panel) (*n* = 6 for each group). Radiances from the peritoneal area of mice in GBC-SD, SGC996 and NOZ cells were shown (right panels) *P < 0.05. The results are shown as mean ± S.D.

To authenticate the *in vitro* findings, we first established subcutaneous tumor mouse model (Figure 2A). When tumors were palpable, IL-6 was injected subcutaneously around the tumor twice a day. The tumor growth increased significantly in the presence of IL-6 (Figure 2A). BrdU staining with subcutaneous mouse tumors demonstrated that IL-6-treated tumor tissues had significantly higher numbers of BrdU-positive nuclei than control group (Figure 2B).

Next, we employed the intraperitoneal dissemination (IPD) mouse model via intraperitoneal (IP) injection to monitor the dissemination potential of GBC cells *in vivo*. We consistently observed that IL-6 treatment had increased overall tumor burden (as measured by radiance) starting at 48 days after cell engraftment (Figure 2C). Collectively, all results above demonstrate that IL-6 promotes GBC cells proliferation and metastasis both *in vitro* and *in vivo*.

### IL-6 promoted GBC metastasis through the regulation of EMT

**Figure 3:**
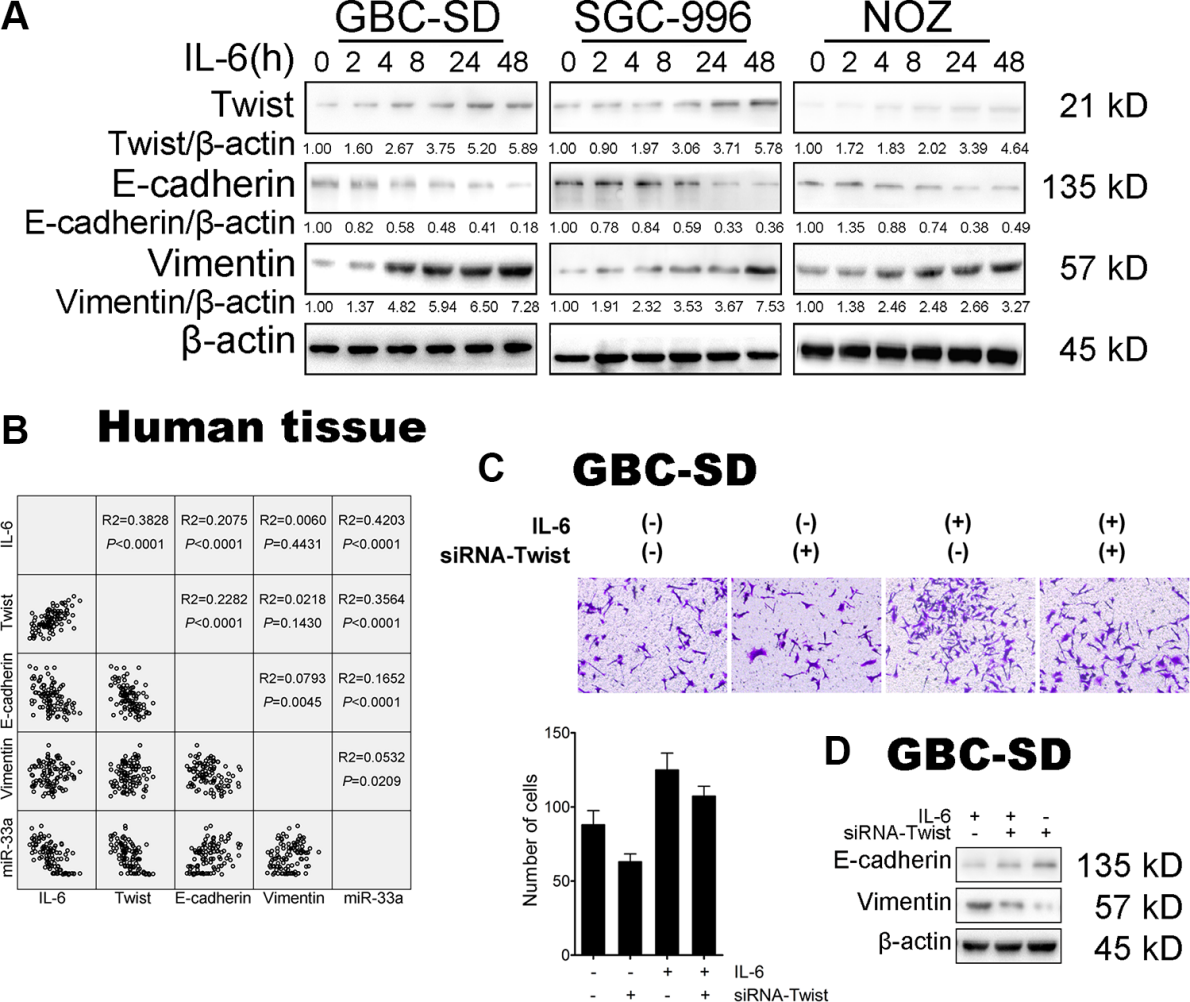
IL-6 induced GBC EMT-mediated metastasis via Twist (**A**) The expression of Twist, E-cadherin and Vimentin in GBC cells after IL-6 treatment. (**B**) Linear regression of IL-6, Twist, E-cadherin, Vimentin mRNA and miR-33a expression in GBC tissues. (**C**) The effect of transfection of siRNA-Twist with IL-6 treatment on migration in GBC-SD cells. Quantitative analysis of migrated cells (lower panels), *P < 0.05. The results are shown as mean ± S.D. (**D**) Western blotting analysis of E-cadherin and Vimentin protein levels in GBC cells after transfection of siRNA-Twist with IL-6 treatment. The results are shown as mean ± S.D.

To further investigate the mechanism of IL-6 on the metastasis of GBC, we detected the expression of EMT-related genes, E-cadherin and Vimentin, both *in vitro* and *in vivo*. Western blotting (Figure 3A) and IF staining (Supplementary Figure S2A) showed that IL-6 decreased the expression of epithelial marker, E-cadherin, while concomitantly increased that of mesenchymal marker, Vimentin, in a time-dependent manner. Immunohistochemistry (IHC) staining showed that the expression of Vimentin protein was enhanced in response to IL-6 treatment in subcutaneous mouse tumors (Supplementary Figure S2B). Reciprocally, the decreased expression of E-cadherin was also observed in IL-6-treated mouse tumor tissues (Supplementary Figure S2B).

### IL-6 induced GBC metastasis via Twist

As Twist has been identified to be activated in EMT-mediated metastasis, we then explored the function of Twist in IL-6-induced GBC metastasis. Western blotting (Figure 3A) and IF staining (Supplementary Figure S2A) showed that Twist protein level was significantly increased in IL-6 treated group compared with control cells. IHC staining also showed the elevated expression of Twist protein in subcutaneous mouse tumor tissues (Supplementary Figure S2C). Surprisingly, Twist mRNA level was only slightly changed in the presence of IL-6 (Supplementary Figure S3A).

To further evaluate the potential relationship between IL-6 and Twist, expression of IL-6 and Twist was analyzed *in vivo*. Statistical analysis of the correlation coefficient revealed a significant positive correlation between the expression of IL-6 and Twist mRNA in 50 human GBC tissues (r^2^ = 0.3828, *P* < 0.001) (Figure 3B).

### Twist is necessary for IL-6-induced GBC cell metastasis

Given that IL-6 could upregulate the expression of Twist, we wondered whether Twist is responsible for the IL-6-mediated malignant phenotypes of GBC-SD cells. Hence, we examined the effect of IL-6 on cell proliferation and metastasis in the absence of Twist. Depletion of Twist by siRNA demonstrated the expected efficiency (Supplementary Figure S3B). IL-6-induced proliferation, migration and metastasis were significantly reversed in GBC cells after Twist siRNA treatment (Supplementary Figure S3C and Figure 3C and Figure 3D). Taken together, these results suggest that Twist is activated in IL-6-induced metastasis, which is essential for the pro-metastasis capability of IL-6 during GBC progression.

### MiR-33a is a direct functional Twist-binding miRNA

The discrepancy between Twist protein and mRNA expression suggests that IL-6-mediated Twist alteration might be mainly regulated at post-transcriptional level, such as miRNA suppression of protein translation. To determine whether there are Twist-binding miRNAs in the IL-6-induced GBC metastasis, we performed comparative miRNA screening of IL-6-treated GBC-SD cell line versus unstimulated group by Affymetrix GeneChip^®^miRNA 3.0 Array (Affymetrix). We further performed bioinformatics analysis by using three miRNA target prediction softwares (TargetScan, miRDB and miRanda) and miRBase to screen out Twist-binding miRNAs among differentially expressed miRNAs.

**Figure 4:**
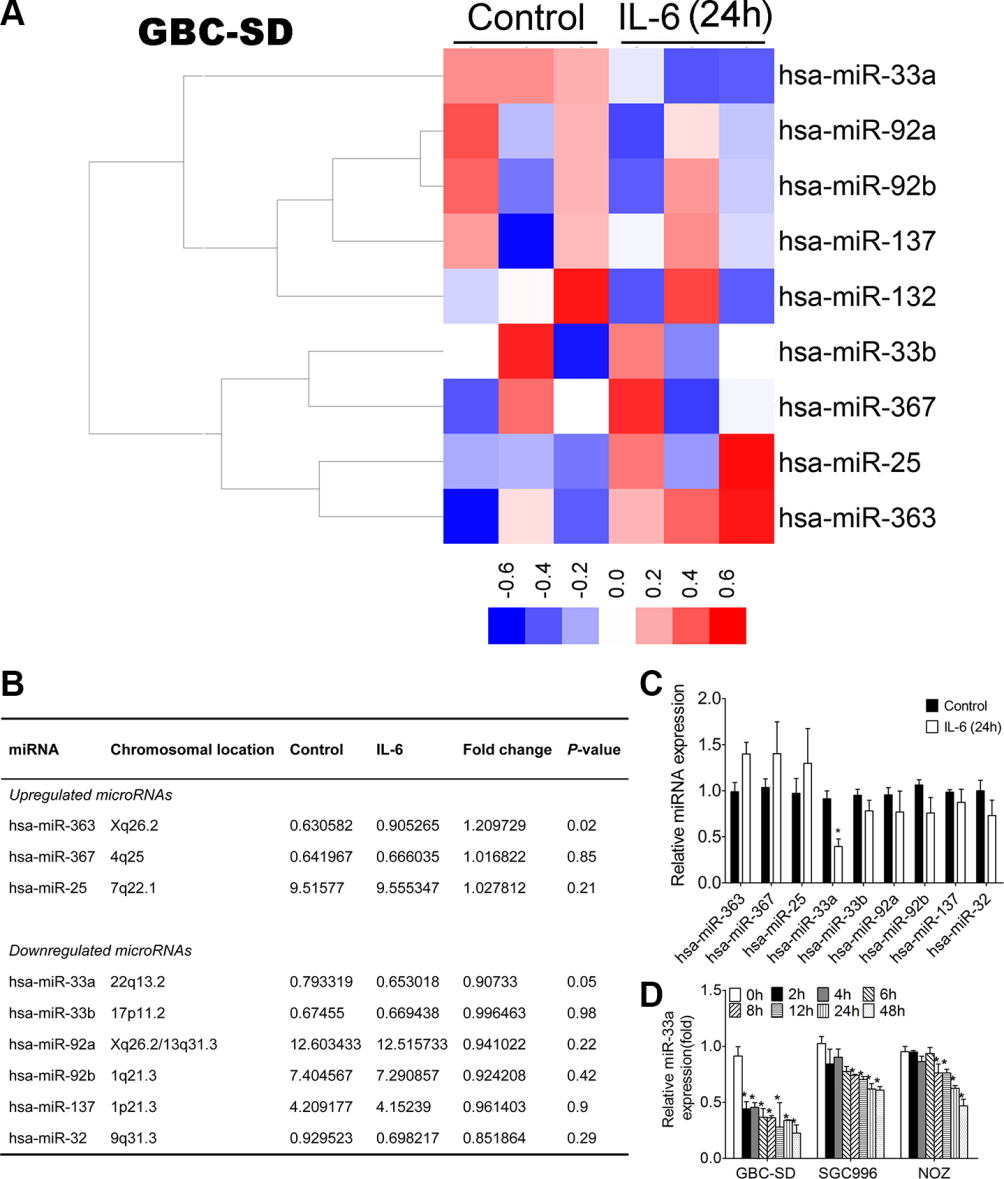
miRNA profilings of IL-6 treated cells and validation of microarray data using qRT-PCR (**A**) Heatmap of miRNAs that targeted Twist clustered in IL-6 treated cells and control (red represents increased expression and blue represents decreased expression). (**B**) The list of changed miRNAs that targeted Twist identified in IL-6 treated cells. (**C**) Validation of miRNAs expression 24 h after IL-6 treatment using qRT-PCR, *P < 0.05. The results are shown as mean ± S.D. (**D**) Validation of miR-33a expression after IL-6 treatment using qRT-PCR, *P < 0.05. The results are shown as mean ± S.D.

Combining the above results, nine putative miRNAs were identified to potentially target the human Twist 3′UTR through multiple binding sites. Among which we found that three miRNAs (miR-363, miR-367, miR-25) were commonly upregulated while six (miR-33a, miR-33b, miR-92a, miR-92b, miR-137, miR-32) were downregulated in IL-6-treated GBC-SD cell line samples compared to the representative controls (Figure 4A and Figure 4B). Considering that the downregulation of miR-33a was much stronger than that of other miRNAs in IL-6 treated GBC-SD cell line (Figure 4C) and few studies have investigated the pathogenesis of this miRNA on GBC, we selected miR-33a for further investigation. Downregulation of miR-33a by IL-6 was further validated in GBC cell lines by qRT-PCR (Figure 4D).

**Figure 5:**
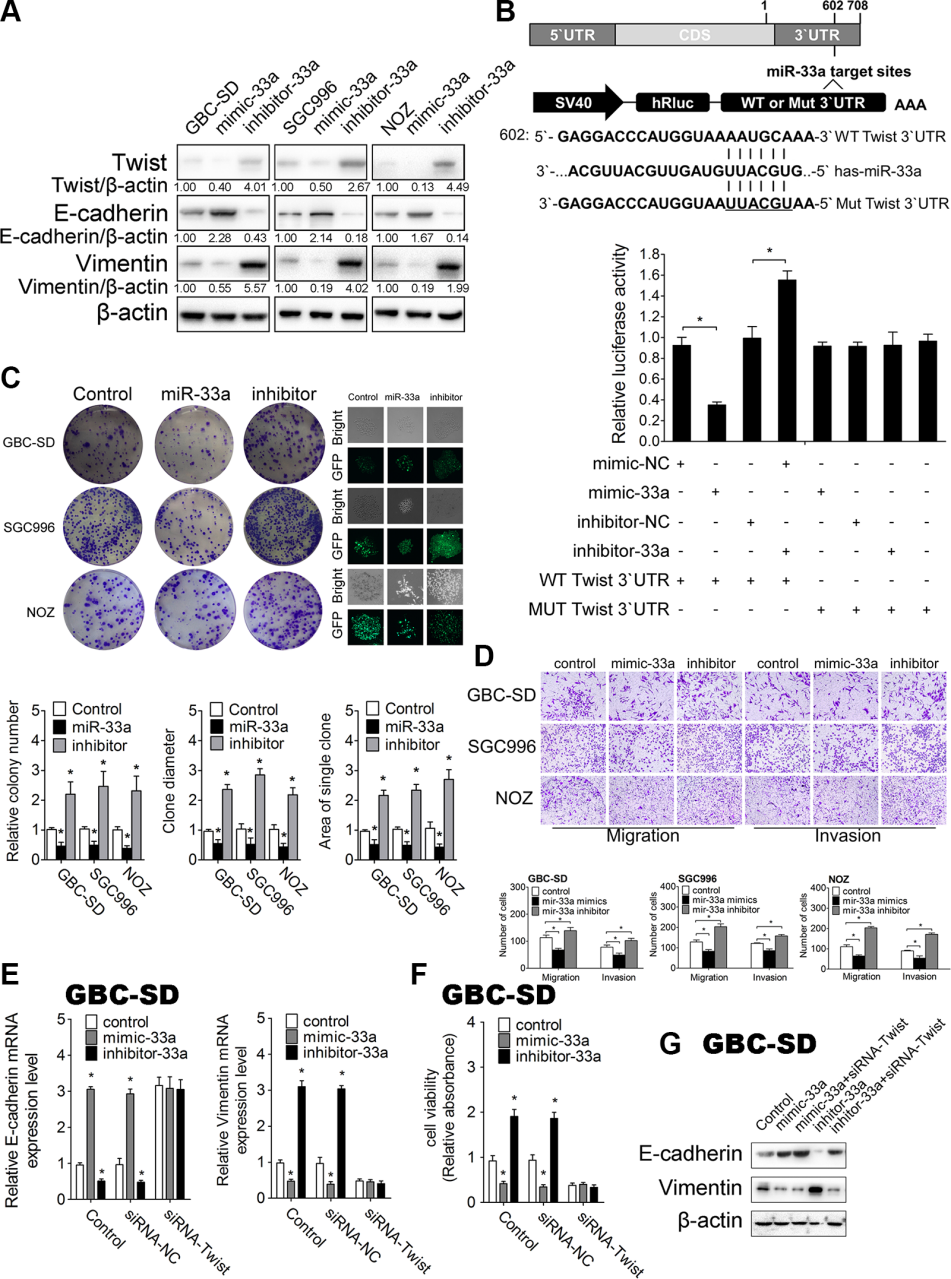
MiR-33a inhibits metastasis of GBC by targeting Twist *in vitro* (**A**) The effect of mimic-33a, inhibitor-33a, or their negative controls on Twist, E-cadherin and Vimentin protein levels in GBC cells detected by western blotting. (**B**) A schematic diagram illustrating the design of luciferase reporters with the WT Twist 3′UTR (WT 3′UTR) or the site-directed mutant Twist 3′UTR (MUT 3′UTR) (upper panel). The effect of miR-33a mimic, inhibitor, or their negative controls on the luciferase activity of WT Twist 3′UTR or MUT Twist 3′UTR reporter in GBC cells (lower panel), *P < 0.05. The results are shown as mean ± S.D. (**C**) The colony formation assays and GFP expression (left lower panel) in GBC cells transfected with control, pcDNA6.2-miR-33a, or pcDNA6.2-miR-33a inhibitor. Quantitative analysis of colony number, clone diameter and area of single clone (right three panels), *P < 0.05. The results are shown as mean ± S.D. (**D**) The effect of mimic-33a, inhibitor-33a, or their negative controls on migration and invasion in GBC cells. Quantitative analysis of migrated cells (lower three panels), *P < 0.05. The results are shown as mean ± S.D. (**E**) qRT-PCR analysis of E-cadherin and Vimentin mRNA levels in GBC cells after cotransfected siRNA-Twist with mimic-33a, inhibitor-33a, or their corresponding negative controls, *P < 0.05. The results are shown as mean ± S.D. (**F**) CCK-8 assay to detect cell viability in GBC cells after cotransfected siRNA-Twist with mimic-33a, inhibitor-33a, or their corresponding negative controls, *P < 0.05. The results are shown as mean ± S.D. (**G**) Western blotting analysis of E-cadherin and Vimentin protein levels in GBC cells after cotransfected siRNA-Twist with mimic-33a, inhibitor-33a, or their corresponding negative controls. CDS, coding sequence; NC, negative control.

To investigate if gain- and loss-of-function of miR-33a exert post- transcriptional regulation on Twist during IL-6-induced GBC metastasis, mimic-33a/inhibitor-33a was transfected to over-express/knockdown miR-33a respectively in GBC cells. Intracellular miR-33a levels were significantly upregulated by mimic-33a treatment and markedly downregulated by inhibitor-33a (Supplementary Figure S4A). Accordingly, Twist protein level was decreased by miR-33a overexpression, and increased by miR-33a knockdown in IL-6 treated GBC cells (Figure 5A), whereas only a mild increase in Twist mRNA level changes was found (Supplementary Figure S4B).

Besides, linear correlation analyses showed that miR-33a expression inversely correlated with the amount of Twist in samples from patients with GBC and their controls (r^2^ = 0.3564, *P* < 0.0001) (Figure 3B).

To further investigate whether miR-33a could directly bind Twist mRNA, we performed luciferase reporter assay. Luciferase reporters that had either a wild-type (WT) Twist 3′ UTR or Twist 3′ UTR containing mutant sequences of the miR-33a binding site were constructed, and co-transfected with miR-33a oligos in HEK293-T cells (Figure 5B). First, we found that mimic-33a substantially inhibited the luciferase reporter activity of the WT Twist 3′ UTR. Second, the luciferase reporter activity of the WT Twist 3′ UTR was significantly increased after reducing the endogenous levels of miR-33a by treating GBC cells with inhibitor-33a. Third, the luciferase reporter activity of Twist with a mutated 3′ UTR was neither repressed nor enhanced by mimic-33a or inhibitor −33a (Figure 5B).

Taken together, all our results reveal that miR-33a directly targets Twist through binding to its 3′UTR during IL-6-induced GBC metastasis.

### Inhibition of IL-6-mediated GBC proliferation and metastasis by miR-33a *in vitro*

Next, we sought to assess the function of miR-33a in GBC biology. We constructed miR-33a and miR-33a-inhibitor expression plasmids under the control of the CMV promoter (pcDNA6.2-miR-33a, and pcDNA6.2-miR-33a-inhibitor, Supplementary Figure S4C). Intracellular miR-33a levels and Twist expression level after plasmids transfection were confirmed by qRT-PCR (Supplementary Figure S4D). Colony-formation assay and CCK-8 assay revealed that the colony formation capability and the percent of live cells were significantly decreased by the transfection of pcDNA6.2-miR-33a but markedly increased by its inhibitor, which suggest the important role of miR-33a in cancer cell growth (Figure 5C and Supplementary Figure S4E). Transwell migration assay showed that miR-33a over-expression significantly decreased cellular migration and invasion (Figure 5D).

To further determine whether miR-33a inhibits IL-6 induced GBC metastasis, we detected EMT biomarkers by Western blotting and qRT-PCR. Consistently, we found that GBC cells transfected with mimic-33a exhibits higher level of E-cadherin and decreasing Vimentin expression, while transfection with inhibitor-33a had the opposite effects (Figure 5A and Supplementary Figure S4F and S4G). Furthermore, IL-6-induced GBC proliferation, migration, Twist expression and the expression of EMT-related proteins were blocked by overexpression of miR-33a (Supplementary Figure S4H–S4J).

To determine whether the effect of miR-33a in GBC metastasis is Twist- dependent, we knocked down Twist in GBC cells and repeated above tests with miR-33a oligos. We found that transfection of Twist siRNA significantly reduced Vimentin mRNA expression and cell viability but promoted E-cadherin mRNA expression (Figure 5E and 5F). However, when we cotransfected mimic-33a and inhibitor-33a with Twist siRNA, the functions of miR-33a oligonucleotides were completely blocked. Both the expression of EMT-related genes and cell viability persistently remained stable (Figure 5E–5G). Cotransfection of Twist siRNA completely blocked miR-33a-induced GBC proliferation and EMT- mediated metastasis, suggesting that the inhibitory effect of miR-33a on GBC metastasis is Twist-dependent. Taken together, our results indicate that miR-33a is inhibited in GBC metastasis through downregulation of Twist *in vitro*.

### Inhibition of IL-6-mediated GBC proliferation and metastasis by miR-33a *in vivo*

**Figure 6:**
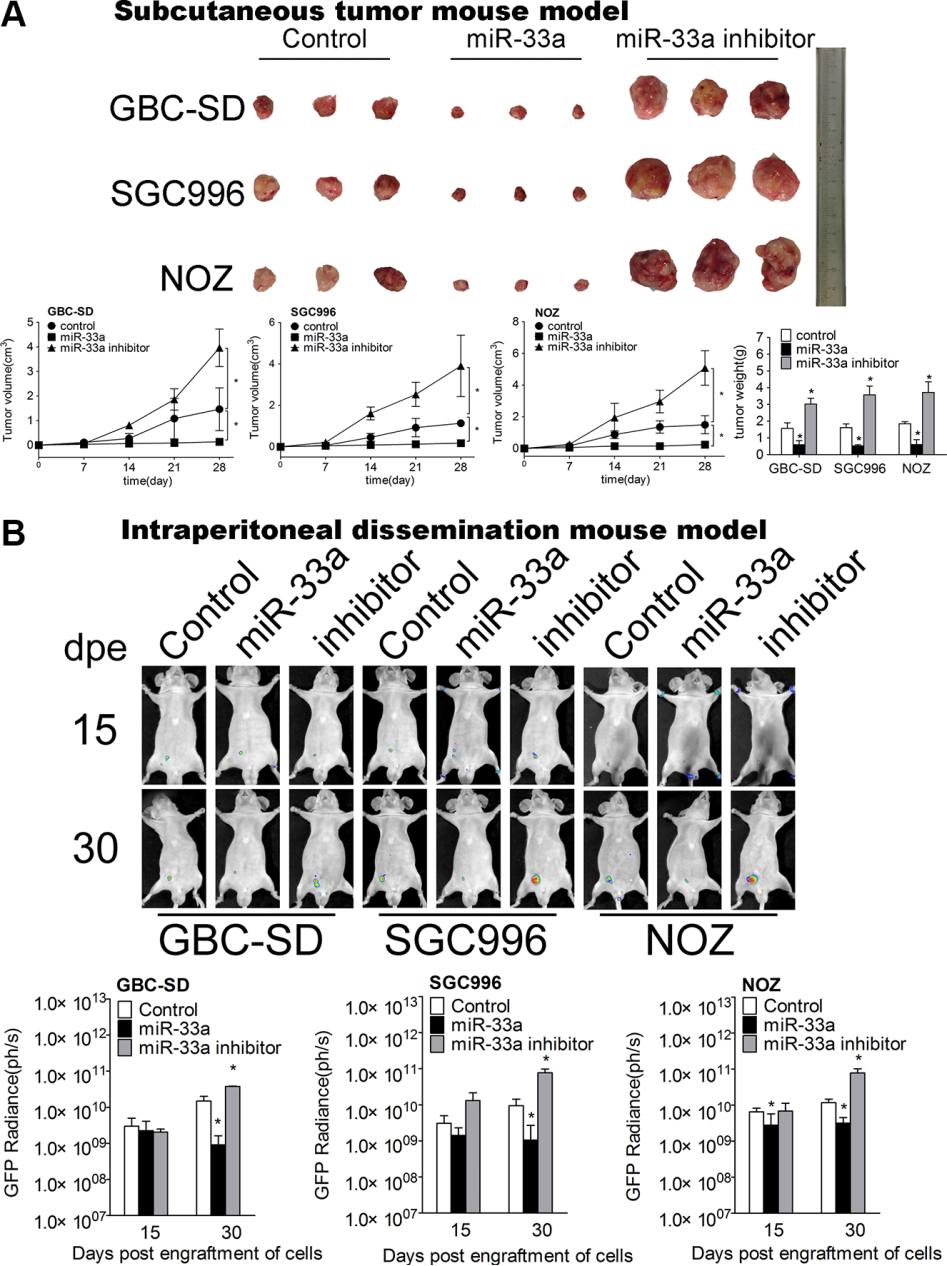
miR-33a inhibits metastasis of GBC *in vivo* (**A**) Mice bearing GBC subcutaneous tumor models after transfection of control, pcDNA6.2-miR-33a, or pcDNA6.2-miR-33a inhibitor (*n* = 6 for each group). The lower panel shows the volume and weight of the tumors, *P < 0.05. The results are shown as mean ± S.D. (**B**) The expression of GFP (right three panels) in mice intraperitoneal dissemination models of GBC-SD, SGC996 and NOZ cells transfected with control, pcDNA6.2-miR-33a, or pcDNA6.2-miR-33a inhibitor. dpe, days post engraftment (*n* = 6 for each group). The results are shown as mean ± S.D.

Next we used subcutaneous tumor mouse model (Figure 6A and Supplementary Figure S5) and IPD model (Figure 6B) to further investigate the effect of miR-33a on GBC metastasis *in vivo*. GBC cells transfected with pcDNA6.2-miR-33a were injected subcutaneously and intraperitoneally respectively. MiR-33a over-expression in GBC cells significantly decreased the mean volume, weight (Figure 6A) and GFP radiance of tumor (Figure 6B and Supplementary Figure S5).

Together, these data highlight a hitherto undefined role of miR-33a in GBC and clarify a unique mechanism of miR-33a-Twist-EMT cascade contributing to GBC progression.

### Decreased expression of miR-33a correlates with poor patient outcome

**Figure 7:**
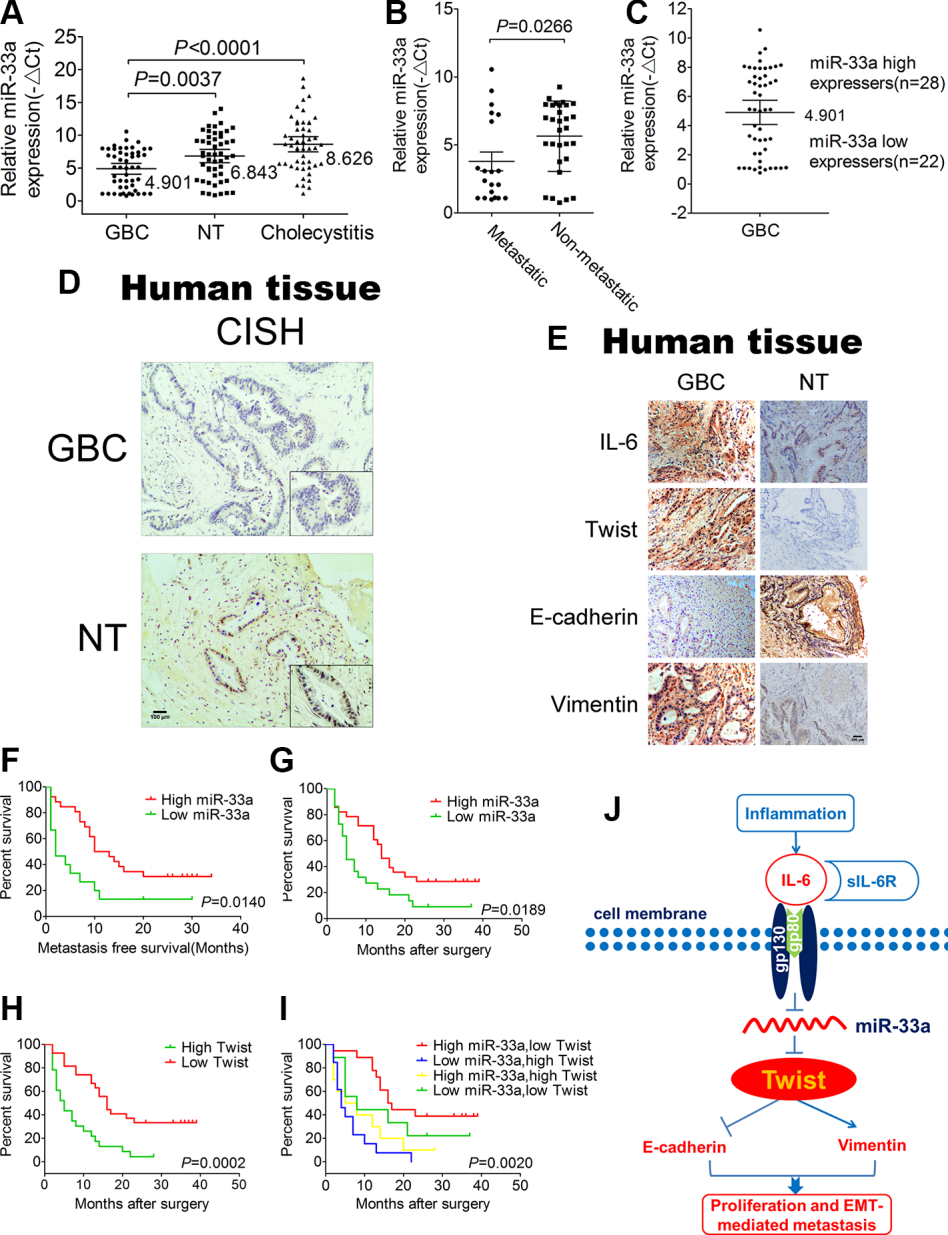
Correlations of miR-33a and pathological characteristics of GBC patients (**A**) The expression of miR-33a in GBC tissues, adjacent normal tissues and cholecystitis tissues. The results are shown as mean ± S.D. (**B**) The expression of miR-33a expression in patients with or without metastasis. The results are shown as mean ± S.D. (**C**) The GBC samples were divided into two groups according to the mean expression of miR-33a. Cases with levels of miR-33a below the mean were miR-33a low expressers (*n* = 22), and those with levels of miR-33a above the mean were miR-33a high expressers (*n* = 28). The results are shown as mean ± S.D. (**D**) *In situ* hybridization to measure the expression of miR-33a in GBC tissues and adjacent normal tissues. (**E**) Representative IL-6, Twist, E-cadherin, and Vimentin expression in GBC tissues and adjacent normal tissues (×200). (**F**) Kaplan–Meier curves for metastasis-free survival of GBC patients between high and low miR-33a expression groups. (**G**) Kaplan–Meier survival curve for GBC patients between high and low miR-33a expression groups. (**H**) Kaplan–Meier curves for GBC patients between high and low Twist expression groups. (**I**) Kaplan–Meier curves for GBC patients between the high miR-33a/low Twist expression group and the low miR-33a/high Twist expression group. A *p* value was generated from all groups together. (**J**) Schematic representation of the function and potential mechanism of miR-33a in GBC. NT, normal tissues.

To assess the clinical relevance of miR-33a in GBC, we analyzed its expression in 50 pairs of clinically and pathologically characterized GBC tissues, case-matched normal tissues and 50 cholecystitis tissues by qRT-PCR. The clinicopathological features were shown in Supplementary Table S1. The expression of miR-33a in GBC tissues (4.901 ± 2.920) was much lower than that of in adjacent normal tissues (6.843 ± 3.584) (*P* < 0.0001) and cholecystitis tissues (8.626 ± 4.092) (*P* = 0.0037) (Figure 7A). GBC patients who developed metastasis showed significantly lower miR-33a expression than those without metastasis (*P* = 0.0266) (Figure 7B), which implies that miR-33a may inhibit GBC metastasis. Using the mean expression value of miR-33a as a cutoff point (4.901) (Figure 7C), the cohort was dichotomized into miR-33a-high (*n* = 28) or miR-33a-low expressers (*n* = 22). Consistent with the above data, *in situ* hybridization results revealed that miR-33a was strongly downregulated in GBC tissues compared with adjacent normal tissues (Figure 7D). In line with our previous results obtained via cell line and animal model experiments, IHC staining with anti-IL-6, Twist, E-cadherin and Vimentin showed similar changes in GBC tissues or adjacent normal tissues (Figure 7E). There was no significant correlation between miR-33a expression and gender, age, HBV infection and peritoneal adhesion as indicated in Supplementary Table S1. However, the miR-33a level inversely correlated with tumor size, degree of differentiation, local invasion, lymph node metastasis and TNM stage (*P* < 0.05) (Supplementary Table S1).

To further analyze the significance of miR-33a in terms of clinical prognosis, Kaplan–Meier survival analysis was performed using patient's overall survival. We observed that low expression of miR-33a is a significant predictor of subsequent metastasis (*P* = 0.0140) and death (*P* = 0.0189) (Figure 7F and 7G). Compared with the high expression group, the median survival time was 5 months in low expression group and 14 months in high expression group (*P* = 0.0189) (Figure 7G). Additionally, the high expression of Twist in GBC also predicted a poor prognosis (Figure 7H). More importantly, those patients with both decreased miR-33a and elevated Twist level exhibited even worse prognosis, suggesting that the combination of two factors provides more prognostic accuracy in comparison with either of them individually (Figure 7I). Furthermore, univariate analysis identified the expression of miR-33a as well as tumor size, degree of differentiation, local invasion, lymph-node metastasis and TNM stage as poor prognosticators for GBC (*P* < 0.05) (Supplementary Table S2). The final multivariate model revealed that reduced miR-33a level in GBC was an independent predictor of shorter survival (HR = 0.286, CI = 0.101–0.811, *P* = 0.019) (Supplementary Table S3).

Taken together, these data support the association between low miR-33a expression and poor patient outcome which elucidates the biological effect of miR-33a on GBC metastasis.

## DISCUSSION

As one of the most aggressive and lethal tumors, GBC has a remarkable tendency for early lymph node metastasis, direct invasion of the liver and tumor recurrence [6, 20, 21]. However, molecular mechanisms mediating GBC progression are poorly understood.

The parallel frequencies of gallstones and GBC in certain populations are the considerable evidence in favor of a direct link between them [22]. Although the pathogenetic mechanisms have not been fully established, gallstones might cause direct mechanical irritation to the surrounding mucosal surface and inflammation [21]. Inflammation can supply inductive molecules to the tumor microenvironment and facilitate angiogenesis, invasion, and metastasis [23–25]. The cytokine IL-6, as one of the most potent metastatic inducers, is required for the development of inflammation-associated cancers [11]. However, there are few reports on animal experiments or clinical specimens with regard to the high expression of IL-6 in GBC.

In our previous study, we validated the high expression of IL-6 in human GBC tissues and identified the relationship between IL-6 and EMT-related factors, patient prognosis in GBC. While due to the complexity and the limited number of tumor tissues, some potential pivotal metastatic inducers may be neglected. In this study, we address this gap in knowledge and confirm proliferation and EMT-related metastasis induced by IL-6 in GBC cell lines, mouse model and clinical specimens.

The available evidence indicates that aberrant activation of EMT is an early step in cancer metastasis [24, 26–28]. Nevertheless, how Twist overexpression confer traits on cancer cells facilitating completion of metastasis still need to be fully elucidated. In this study, we found the discrepancy between Twist protein and mRNA expression after IL-6 treatment which suggested that Twist might be mainly regulated at post-transcriptional level, such as miRNA suppression of protein translation. However, we also found the discrepancy between the expression of Twist mRNA in GBC cell lines and in human GBC tissues (Supplementary Figure S4B and Figure 3B). A general model for gene regulation by miRNAs holds that partial base-pairing between a miRNA and its target mRNA results in translational repression without destabilization of the mRNA [29]. However, recent studies provide overwhelming evidence that the miRNA-bound mRNA is also subject to degradation [30, 31]. These studies suggested that miRNA-supported mRNA degradation and translation repression may occur independently of each other [32–34]. The other study suggested translational inhibition may precede destabilization of miRNA targets [30]. Regulation by miRNAs can result in destabilization of target mRNAs containing sites of imperfect complementarity *in vivo*. However, the *in vitro* systems may not be competent for the mRNA destabilization activity [30].

In our study, miRNA profiling was performed and miR-33a was identified as a commonly decreased miRNA in IL-6 treated GBC cells. miR-33a was first reported to inhibit fatty acid metabolism and insulin signaling [35]. The tumor relevant studies indicated miR-33a functions as a tumor suppressor and the potential effect on cancer treatment was mentioned [36–39]. Moreover, miR-33a was found to contribute to the chemotherapeutic resistance of osteosarcoma and pancreatic cancer cells [40, 41]. In spite of the growing evidence highlighting the importance in various cancers, none of the previous studies have systematically investigated the role of the miR-33a in GBC progression. Herein, we have identified a single miRNA-miR-33a in IL-6 treated GBC cells, which is inhibited in GBC metastasis through downregulation of Twist. We show that miR-33a inhibited cell proliferation, cell migration and invasion of GBC, consistent with its common downregulation in human GBC tissues. The Low expression of miR-33a was also correlated with local invasion, lymph node metastasis and poor prognosis. Our present data further demonstrate that miR-33a- mediated inhibition of Twist is dependent on a conversed motif in the 3′UTR of Twist. The reducing of miR-33a is responsible for IL-6-induced metastasis followed by the activation of Twist, which may further reinforce IL-6-induced GBC progression (Figure 7J). Intriguingly, the functions of miR-33a oligonucleotides were completely blocked after the cotransfection of mimic-33/inhibitor-33a and Twist siRNA which further emphasizing the multiple roles of miR-33a involving GBC progression through binding Twist.

In conclusion, this study provides important clues underpinning the pivotal role of miR-33a on IL-6-induced GBC progression by directly binding the transcription of Twist. Our discovery predicts potential diagnostic and prognostic value for this miRNA as a biomarker. These results may also have implications for the clinical management of patients with metastatic disease.

## MATERIALS AND METHODS

Additional detailed description of the materials and methods can be found in the online supplementary materials and methods section.

## SUPPLEMENTARY MATERIALS


