# Supplementary material for: The microRNA miR-33a suppresses IL-6-induced tumor progression by binding Twist in gallbladder cancer
Source: Oncotarget. 2016 Oct 15;7(48):78640–52. doi: 10.18632/oncotarget.12693 (PMC5346666; doi:10.18632/oncotarget.12693)
Supplement: Supplementary file 1 [file oncotarget-07-78640-s001.pdf]

The microRNA miR-33a suppresses IL-6-induced tumor progression by binding Twist in gallbladder cancer

Supplementary Materials

MATERIALS AND METHODS

Tissue samples

A total of 50 GBC specimens along with adjacent normal tissues were randomly retrieved from GBC patients who underwent radical cholecystectomy (between 2007 and 2009 in Eastern Hepatobiliary Surgery Hospital, Second Military Medical University, Shanghai, China.), and 50 cholecystitis tissues were obtained from patients who were diagnosed with benign diseases and underwent surgical resection (between 2013 and 2014 at the Department of General Surgery, Xinhua Hospital, School of Medicine, Shanghai Jiao Tong University, China.). The clinicopathological features were shown in Supplementary Table S1. All GBC patients were retrospectively followed up until September 2013, none of whom received chemotherapy or radiotherapy after surgery.

Cell lines

Three human gallbladder cancer cell lines were used in this study: GBC-SD (Shanghai cellbank of Chinese Academy of Sciences, China), SGC996 (Academy of Life Science, Tongji University, China) and NOZ (Health Science Research Resources Bank, Osaka, Japan). GBC-SD and SGC-996 were cultured in Dulbecco's modified Eagle's medium (DMEM) (Gibco, USA) containing 10% fetal bovine (FBS) (Gibco, USA). NOZ cells were cultured in Williams E medium (Gibco, USA) supplemented with 10% FBS.

Ethics statement

All experimental procedures were approved by the Hospital's Protection of Human Subjects Committee; Xinhua Hospital affiliated with Shanghai Jiaotong University, the Eastern Hepatic Biliary Hospital affiliated with the Second Military Medicine University. Written informed consents were obtained for each patient and the study protocol conformed to the ethical guidelines of the 1975 Declaration of Helsinki as reflected in a priori approval by the appropriate institutional review committee. All animals in our study received humane care according to the criteria outlined in the "Guide for the Care and Use of Laboratory Animals" prepared by the National Academy of Sciences and published by the National Institutes of Health (NIH publication 86–23 revised 1985).

AffymetrixGeneChip®miRNA 3.0 Array and data analysis

The GeneChip®miRNA 3.0 Array probes were synthesized according to the Sanger miRBase V18. We selected IL-6 treated group and control group for miRNA array analysis. The cells were harvested 24 h after treatment of IL-6/ saline. Microarray experiments were conducted according to the manufacturer's instructions. Briefly, 1 µg of total RNA was labelled using the Flash Tag Biotin Labelling Kit (Affymetrix, USA). The labelling reaction was hybridised with the miRNA Array in an Affymetrix Hybridization Oven 640 (Affymetrix, USA) at 48°C rotating at 60 rpm for 16 h. The arrays were stained in the Fluidics Station 450 using fluidics script FS450_0003 (Affymetrix, USA) and then scanned on the GeneChip® Scanner 3000 (Affymetrix, USA). MicroRNA probe outliers were defined per the manufacturer's instructions (Affymetrix, USA), and further analysis included data summarization, normalization and quality control using the web based miRNA QC Tool software (www.affymetrix.com). The raw data were processed in the following sequence: background detection was followed by RMA global background correlation, quantile normalization, median estimation and log2- transformation using the miRNA QC software tool (Affymetrix, USA). Finally, we performed tree visualisation using Cluster 3.0 and Treeview 1.0.4.ext.

Recombinant interleukin-6 treatment

Recombinant human IL-6 was purchased from PeproTech (Rocky Hill, USA). GBC-SD, SGC996 and NOZ cells were cultured in plates to 80% confluence. After cells had attached, media were replenished with DMEM containing no serum and incubated for an additional 24 h to synchronize cells in a non-activating and non-proliferating phase. Cells were all pretreated with IL-6 for the next *in vitro* experiment.

BrdU staining and feeding

After IL-6 treatment for 24 h, BrdU (Sigma-Aldrich, USA) staining with cultured cells was performed according to manufacturer's instructions. Stained cells were detected by immunofluorescence staining assay with BrdU antibody (1:200, Abcam, USA) and visualized under fluorescent microscope (Olympus, Japan).

In vivo test, mice with or without IL-6 treament were injected with 10–100 mg/kg BrdU in saline intraperitoneally (IP). Animals were sacrificed 24 h after BrdU injection. BrdU-labeled cells were counted under fluorescence microscope (Olympus, Japan).

Cell migration and invasion assays

For *in vitro* wound-healing assay, starved cells were treated with IL-6, and a cell-free area was created by scratching the monolayer with a 200 μl pipette tip. Cell migration into the wound area was monitored in serum-free medium. After 24 h and 48 h, photographs were taken using an inverted microscope (Olympus, Japan).

Cell migration and invasion were further determined using 8 μm transwell chambers (Millipore, USA) coated with/without Matrigel (BD, USA). 4×10^4 GBC cells in 0.2 ml serum-free medium were added to the upper chamber containing a non-coated/ Matrigel-coated membrane. The lower compartment contained 0.7 ml basal medium containing 10% FBS. After 24 h incubated at 37°C in a 5% CO_2 humidified incubator, cells located on the upper surface were removed using a cotton swab, and the cells on the lower surface were fixed with Methanol, stained with crystal violet, and scored visually in 5 random fields using a light microscope (using the 10 × objective lens, Olympus, Japan).

Immunohistochemistry (IHC), Immunofluorescence (IF) staining and Western blotting

The samples prepared from paraffin-embedded block were rehydrated and then incubated in 3% hydrogen peroxide for 15 min to block endogenous peroxidase. For antigen retrieval, samples were boiled in pressure cooker for 10 min. Nonspecific binding was blocked with 10% normal goat serum for 20 min at 37°C. The samples were then incubated at 4°C overnight with primary antibodies against IL-6 (ab6672; Abcam, USA), Twist (ab50887; Abcam, USA), E-cadherin (sc-7870; Santa Cruz Biotechnology, USA) and Vimentin (sc-5565; USA). The sections were treated with secondary antibody for 30 minutes at room temperature and stained with diaminobenzidine (DAB) until brown granules appeared. The sections were counterstained with hematoxylin for 2 minutes at room temperature. Sections were blindly evaluated by two pathologists with light microscopy. A semi-quantitative scoring criterion for IHC was used, in which expression was determined based on the percentage of positive cells and staining intensity. Scores were interpreted as follows: The final score was the outcome of protein expression rate and intensity, graded as"-" for 0 point, "+" for 1–2 points, "++" for 3–4 points, and "+++" for 5–6 points. Immunoreactivity "±" were categorized as over-expression and "++/+++" as under-expression for statistical analyses.

Cells were fixed in a solution of 4% formaldehyde for 4 h, washed in 0.1% Triton X-100 (PBST) for 15min, and blocked in 5% goat serum for 1 h. Primary antibodies were diluted in blocking solution and incubated overnight at 4°C. Cells were washed and incubated in secondary antibody (Abcam, USA) for 1.5 h at 37°C. Immunofluorescence was analyzed using fluorescence microscopy (Olympus, Japan).

Cells were lysed in RIPA sample buffer (Beyotime, China) supplemented with protease inhibitors (Complete, EDTA-free; Roche, USA) and PMSF (Beyotime, China). The protein concentration was measured using a BCA Protein Assay kit (Beyotime, China). Cell lysates (80 μg/well) were electrophoresed on 8–12% polyacrylamide gels (Bio-Rad, USA) and transferred to polyvinylidene difluoride (PVDF) membranes (Whatman, Germany). The membranes were blocked with a 5–10% skim milk solution and incubated with anti-IL-6 (ab6672; Abcam, USA), anti-Twist(ab50887; Abcam, USA), anti-E-cadherin (sc-7870; Santa Cruz Biotechnology, USA), anti-Vimentin (sc-5565; Santa Cruz Biotechnology, USA) and anti-β-actin antibodies (#4970; Cell Signaling Technology, USA)respectively, at 4°C overnight. This was followed by incubation with goat anti-rabbit/ anti-mouse secondary antibody conjugated with horseradish peroxidase (1:1000; Abcam, USA). The stain was visualized by enhanced chemiluminescent (ECL) detection reagent from Millpore (Rockford, USA). Images were obtained and optical densities of the bands were quantified with Gel Doc 2000 (BioRad, USA).

qRT-PCR for mRNA and miR expression

Total RNA purified from cells and tumor tissues with the Trizol reagent (Takara, Japan) was reversely transcribed to form cDNA using PrimeScript™ RT Master Mix (TaKaRa, Japan), which was subjected to SYBR Green-based (Takara, Japan) qRT-PCR analysis. Reverse transcription and cDNA amplification were performed using the SYBR® PrimeScript™ miRNA RT-PCR Kit (Takara, Japan). The primers for miR-33a and U6 were purchased from Takara, Japan.

The primer sequences were as follows:

β-actin	forward, 5′-CTGGGACGACATGGAGAAAA-3′
	reverse, 5′-AAGGAAGGCTGGAAGAGTGC-3′
IL-6	forward, 5′-CCACACAGACAGCCACTCAC-3′
	reverse, 5′-GATGATTTTCACCAGGCAAGTC-3′
Twist	forward, 5′-AGTCCGCAGTCTTACGAGGAG-3′
	reverse, 5′-GACCTGGTAGAGGAAGTCGATG-3′
E-cadherin	forward, 5′-GTCTCTCTCACCACCTCCACAG-3′
	reverse, 5′-CTCGGACACTTCCACTCTCTTT-3′
Vimentin	forward, 5′-GAAGAGAACTTTGCCGTTGAAG-3′
	reverse, 5′-GAAGGTGACGAGCCATTTC-3′

U6 and β-actin were used as references for miRNAs and RNAs, respectively. The term −ΔCt was used to describe the expression level of mRNA. The $2^{-\Delta\Delta Ct}$ method was used to quantify the relative levels of gene expression. The expression was subsequently divided into lower expression and higher expression groups based on mRNA levels greater or less than the mean.

Mimics, inhibitor and siRNA transfection

Mimics-33a (GenePharma, China) , mimic control, inhibitor-33a (GenePharma, China), inhibitor control, small interfering RNA (siRNA) targeting human Twist (sequence 5′- GCUGAGCAAGAUUCAGACCCUTT -3′, GenePharma, China) and siRNA negative control (100 nM) were transfected into GBC cells using Lipofectamine™ 2000 (Invitrogen, USA). Total RNA was harvested at 24 h after transfection, while total protein was harvested 48 h after transfection.

Cell viability assays

For colony-formation assay, cells were seeded onto 6-well plates (Corning, USA) at a density of 200 cells/ml. After adherence, the cells were stimulated with IL-6 for 48 h and then cultured for 15 days. Thereafter, the cells were fixed with 10% formalin and stained with 0.1% crystal violet (Sigma-Aldrich, USA). For CCK8 test, cells were seeded in 100 μl of growth medium at a density of 3×10^3 cells per well in 96-well plates, followed by IL-6 stimulation (0, 2, 4, 6, 8, 12, 24, 48 h). 10 μl CCK8 solution was added per well 2-4 h before the end of incubation at 37°C. Cell viability was measured with a microplate reader (BioTek, USA) at an absorbance of 450 nm. The values for transfected and/or IL-6 stimulated samples are reported as percent of live cells compared to control samples, which were considered as 100% or 1. Transfected cells were performed according to above instructions

MiR-33a Twist 3′UTR reporter

HEK293-T cells (5×10^4 cells per well) were plated in a 24-well plate and then co-transfected with 50 mM of mimics-33a, inhibitor-33a or a microRNA control, 50 ng of either pGL3-Twist-3`UTR-WT or pGL3-Twist-3`UTR-MUT (Promega, USA) using LipofectamineTM 2000 (Invitrogen, USA). HEK293-T cells were collected 48 h after transfection and analyzed using the dual-luciferase reporter assay system (Promega, USA). Expression of Renilla luciferase was co-transfected as an internal control.

In Situ Hybridization (ISH) of miRNA

ISH was performed using the miRCURY LNA™ Detection probe hsa-miR-33a (Exiqon, Vedbaek, Denmark) and the microRNA ISH Optimization Kit (Exiqon, Vedbaek, Denmark) according to the manufacturer's instructions. For paraffin imbedded tissue, after deparaffinization and rehydration, the samples were treated with peroxidase-quenching solution; proteinase K was added to digest tissues before prehybridization and hybridization, which were carried out at 55°C for 30 min and 4 h, respectively. Then streptavidin–HRP was used to react with the bound biotin-labeled probe. The signal was further amplified using Tyramide signal amplification TSA amplification kit (Perkin Elmer, USA). Finally, the signal was revealed with UltraVision One polymer and AEC chromogen (Thermo Fisher Scientific, USA).

AffymetrixGeneChip®miRNA 3.0 Array and data analysis

The GeneChip®miRNA 3.0 Array probes were synthesized according to the Sanger miRBase V18. We selected IL-6 treated group and control group for miRNA array analysis. Microarray experiments were conducted according to the manufacturer's instructions. Briefly, 1 μg of total RNA was labelled using the Flash Tag Biotin Labelling Kit (Affymetrix, USA). The labelling reaction

was hybridised with the miRNA Array in an Affymetrix Hybridization Oven 640 (Affymetrix, USA) at 48°C rotating at 60 rpm for 16 h. The arrays were stained in the Fluidics Station 450 using fluidics script FS450_0003 (Affymetrix, USA) and then scanned on the GeneChip® Scanner 3000 (Affymetrix, USA). MicroRNA probe outliers were defined per the manufacturer's instructions (Affymetrix, USA), and further analysis included data summarization, normalization and quality control using the web based miRNA QC Tool software (www.affymetrix.com). The raw data were processed in the following sequence: background detection was followed by RMA global background correlation, quantile normalization, median estimation and log2- transformation using the miRNA QC software tool (Affymetrix, USA). Finally, we performed tree visualisation using Cluster 3.0 and Treeview 1.0.4.ext.

Development of firefly luciferase and GFP expressing cell lines

Cells were co-transfected with the pGL4.13 [luc2/SV40] Vector (Promega, USA), pcDNA6.2-GW/EmGFP empty vector, pcDNA6.2-GW/ EmGFP–miR-33a and pcDNA6.2-GW/EmGFP–miR-33a inhibitor (Ruisai, China) using lipofectamine™ 2000 (Invitrogen, USA) according to the manufacturer's instructions. 24 hours after transfection, cells were split 1:6 and plated in the presence of 3 mg/ml (GBC-SD, SGC996) or 6 mg/ml (NOZ) Geneticin (G418) (Invitrogen, USA), 5 μg/μl Blasticidin S (Invitrogen, USA) respectively for 2–3 weeks. Colonies were selected by limiting dilution analysis (LDA). Selected clones were expanded and used in animal studies.

Flow cytometry analysis

The expression profiles of CD133 and CD44 in cultured cells were analyzed by flow cytometry. Briefly, 1×10^7 cells were incubated with 100 μl of 1% BSA in PBS containing 10μl of CD16/CD32 (eBioscience) for 30 min on ice to block unspecific interaction, then labeled with FITC-conjugated anti-CD133 (MiltenyiBiotec, Germany), PE conjugated anti-CD44 (MiltenyiBiotec, Germany) for 10 minutes at 2–8°C. Labeled cells were resuspended in PBS with 1% FBS, and analyzed by flow cytometer (BD, USA).

The expression profiles of Edu proliferation in cultured cells were analyzed by flow cytometry. EdU (Invitrogen, USA) were added to the culture medium at 10 μM for 1–2 hours. 1×10^7 cells were incubated with 100 μl of Click-iT® fixative for 15 minutes at room temperature. Then, cells were resuspended in 100 μl of 1 × Click-iT®saponin-based permeabilization and incubate the cells for 15 minutes. Cells were incubated in 100 μl of 1 × Click-iT®saponin-based permeabilization for 15 minutes. Cells were resuspended in 100 μl of 1 × Click-iT®saponin-based permeabilization for analyzing the cells on a flow cytometer.

Animal models

Subcutaneous tumor models

Approximately 5×10^6 cells were injected subcutaneously into the left hind limb of 4–6-week-old nude mice (Shanghai Laboratory Animal Center of the Chinese Academy of Sciences, China). When tumors were palpable, animals were divided into two groups which were injected subcutaneously around the tumor with either saline, or mouse recombinant IL-6 dissolved in saline (200 ng/200 μl/ mouse) twice a day. Tumor size was measured every other day using an external caliper. Tumor volumes (V) were determined by the formula $V = ab^2/2$, where a is the larger and b is the smaller of the two dimensions. The standard for tumor formation was the diameter of tumors > 0.5 cm. Tumor-bearing mice were sacrificed 5 weeks after tumor inoculation, and the tumors were removed, weighed, and fixed by 10% neutral formalin and embedded in paraffin for Haematoxylin&Eosin (HE) staining and IHC. Seven days after virus injection, tumors were excised, fixed in 10% neutral buffered formalin and paraffin embedded.

Intraperitoneal dissemination (IPD) models

Approximately 5×10^6/200 ml cells expressing firefly luciferase/GFP were injected into the peritoneal cavity of 4–6 week-old nude mice. Seven or nine days after cell implantation, engraftment of luciferase expressing cancer cells was determined by bioluminescent and GFP imaging using the NightOWL LB 983 *in vivo* Imaging System (Berthold, Germany). Mice with detectable luciferase/GFP expression in the intraperitoneal (IP) cavity were then randomly distributed into the various treatment cohorts. All treatment regimens started 5–6 days after cell implantation.

Bioluminescence and GFP *in vivo* imaging

Bioluminescent signals from tumors were detected by IP administration of D-luciferin substrate (150 mg/kg). Five minutes after substrate injection, mice were anesthetize using isofluorane and imaged 5–10 minutes after injection of substrate using the NightOWL LB 983 *in vivo* Imaging System (Berthold, Germany). A luciferin kinetic curve was used to determine the peak luciferase expression time for each tumor model. Mice labled GFP were anesthetize using isofluorane and imaged using the NightOWL LB 983 *in vivo* Imaging System (Berthold, Germany).Data were analyzed based on radiance (ph/s) emitted from the IP cavity of each mice.

Statistical analyses

All data are presented as means± SD and were analyzed using Prism 5.0 software (GraphPad). The significance of the observed differences was determined with Student's *t*-test or the x^2 test. For all other analyses multiple comparisons were made using the analysis of ANOVA. Univariate analyses were performed to investigate clinicopathological and molecular characteristics according to the miR-33a expression level; a chi-square test or Fisher's exact test was used for categorical data. In survival analysis, the Kaplan–Meier method and log-rank test were used to assess the survival time distribution. Cox proportional hazards regression models were used to compute mortality hazard ratios (HR) according to the miR-33a expression status. The relationships among the IL-6 RNA, Twist RNA and miR-33a were analyzed by correlation coefficients and linear regression analysis. $P < 0.05$ was considered to be statistically significant.

Supplementary Table S1: Correlation between clinicopathological features and the expression of miR-33a in GBC

variables	miR-33a low expression group (*N* = 22)	miR-33a high expression group (*N* = 28)	*P*
gender			
male	8	5	0.139
female	14	23	
Age (years)			
< 60	12	13	0.569
≥ 60	10	15	
Tumor size (cm)			
< 5	18	9	**0.000**
≥ 5	4	19	
HBV infection			
Yes	2	1	0.829
No	20	27	
Peritoneal adhesion			
Yes	3	1	0.437
No	19	27	
Degree of differentiation			
Well and moderately differentiated	7	25	**0.000**
Poorly differentiated	15	3	
Local invasion			
positive	16	8	**0.002**
negative	6	20	
Lymph-node metastasis			
positive	14	6	**0.002**
negative	8	22	
TNM stage			
I–II	4	19	**0.000**
III–IV	18	9	

SupplementaryTable S2: Univariate analysis for prognostic factors of GBC

variables	group	case (*n*)	average survival time (months) 95%CI	*P*
gender	male	13	9.692 (4.966–14.419)	0.093
	female	37	16.757 (12.304–21.209)	
Age (years)	< 60	25	14.640 (9.596–19.684)	0.867
	≥ 60	25	15.240 (10.039–20.441)	
Tumor size (cm)	< 5	27	9.704 (6.005–13.402)	**0.003**
	≥ 5	23	21.130 (15.492–26.769)	
HBV infection	Yes	3	17.000 (4.569–29.431)	0.529
	No	47	14.681 (10.952–18.409)	
Peritoneal adhesion	Yes	4	8.250 (2.084–14.416)	0.225
	No	46	15.630 (11.711–19.550)	
Degree of differentiation	Well and moderately differentiated	32	19.625 (14.724–24.526)	**0.000**
	Poorly differentiated	18	6.889 (4.364–9.414)	
Local invasion	positive	24	8.500 (5.546–11.454)	**0.001**
	negative	26	20.577 (15.144–26.010)	
Lymph-node metastasis	positive	20	7.950 (4.707–11.193)	**0.002**
	negative	30	19.333 (14.413–24.254)	
TNM stage	I-II	23	22.130 (16.312–27.949)	**0.000**
	III-IV	27	8.519 (5.843–11.194)	
miR-33a	high expression	28	18.821 (13.703–23.940)	**0.017**
	low expression	22	10.045 (5.772–14.319)	

Supplementary Table S3: Multivariate analysis for prognostic factors of GBC

variables	HR	95%CI	***P***
gender			
male<br>female	0.634	0.304–1.324	0.225
Age (years)			
< 60<br>≥ 60	0.514	0.251–1.051	0.068
Tumor size (cm)			
< 5<br>≥ 5	0.229	0.096–0.543	**0.001**
HBV infection			
Yes<br>No	1	0.245–5.493	0.852
Peritoneal adhesion			
Yes<br>No	0.718	0.219–2.353	0.585
Degree of differentiation			
Well and moderately differentiated<br>Poorly differentiated	4.35	1.531–12.356	**0.006**
Local invasion			
positive<br>negative	0.905	0.089–1.356	0.128
Lymph-node metastasis			
positive<br>negative	0.905	0.324–2.527	0.849
TNM stage			
I–II<br>III–IV	1.71	0.355–8.230	0.503
miR-33a expression			
high<br>low	0.286	0.101–0.811	**0.019**

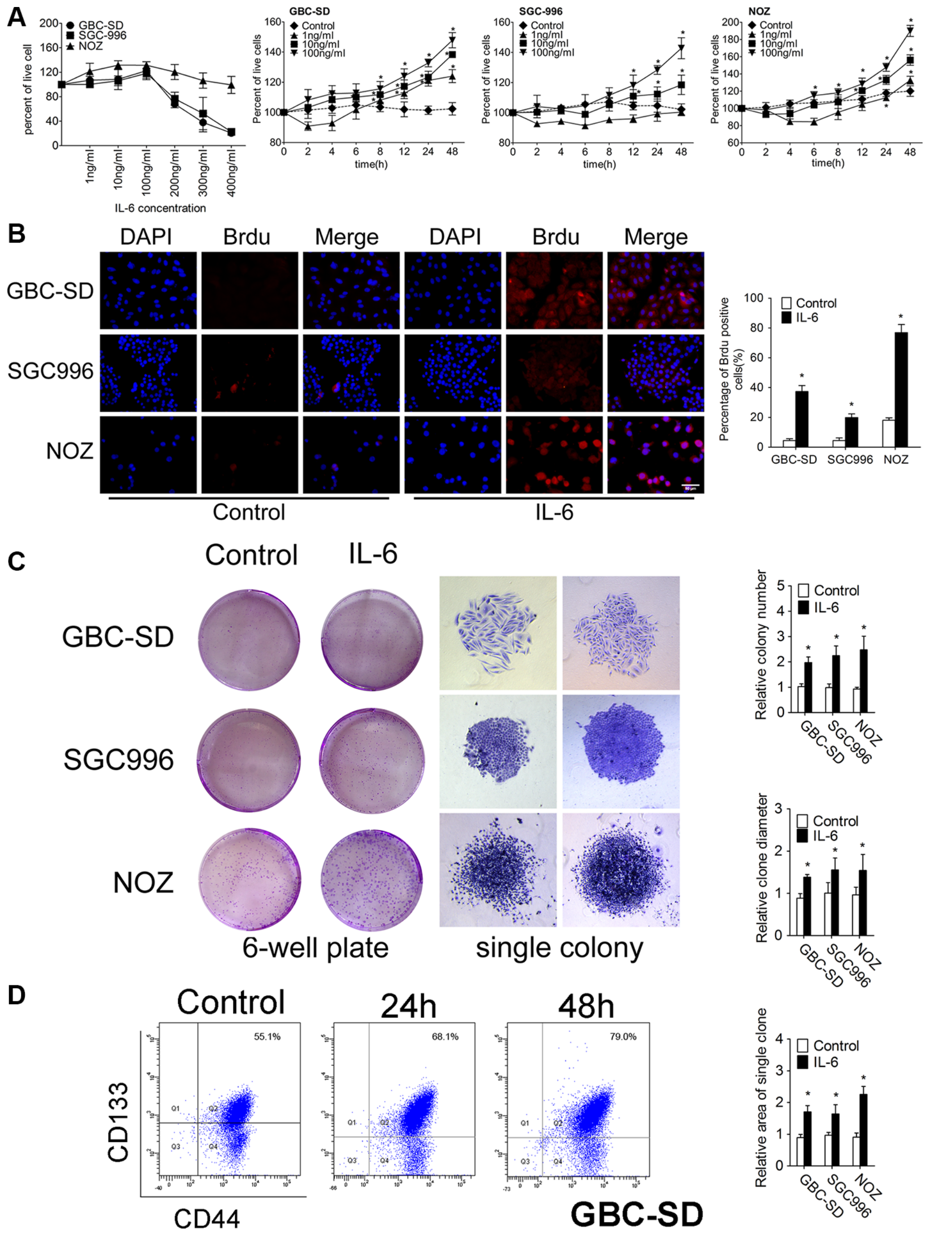

Supplementary Figure S1: IL-6 promotes the proliferation of GBC *in vitro*. (**A**) CKK-8 assay to detect the cell viability of GBC cells with/without IL-6 treatment, $*P < 0.05$. The results are shown as mean ± S.D. (**B**) Immunofluorescence analyzed the Brdu positive cells after IL-6 treatment (×200) (Left panel). Quantitative analysis of percentage of Brdu positive cells (Right panel), $*P < 0.05$. The results are shown as mean ± S.D. (**C**) The colony formation assays in GBC cells after IL-6 treatment (Left panel). Quantitative analysis of colony number, clone diameter and area of single clone (Right three panel), $*P < 0.05$. The results are shown as mean ± S.D. (**D**) Expression profiles of $CD133^+$ and $CD44^+$ GBC cells after IL-6 treatment.

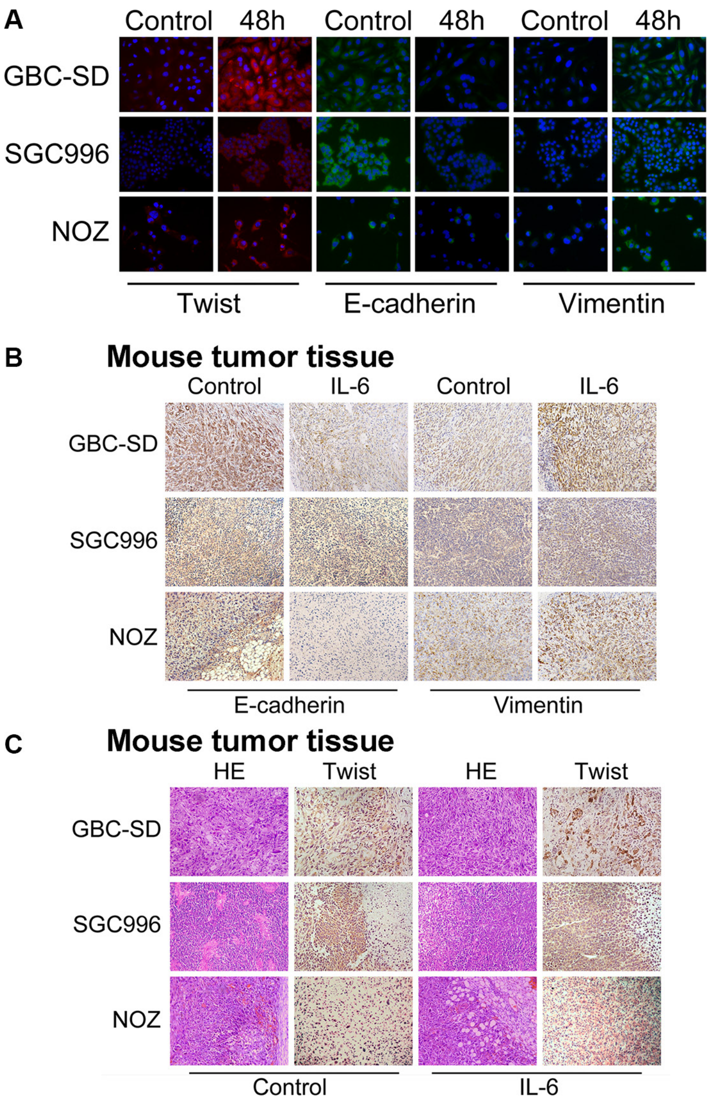

Supplementary Figure S2: IL-6 induced EMT-mediated metastasis *in vitro* and *in vivo*. (**A**) Immunofluorescence analyzed the expression of Twist, E-cadherin and Vimentin in GBC cells after IL-6 treatment (×200). (**B**) Immunohistochemistry staining with E-cadherin and Vimentin in subcutaneous tumor tissues. (**C**) Immunohistochemistry staining with Twist in subcutaneous tumor tissues.

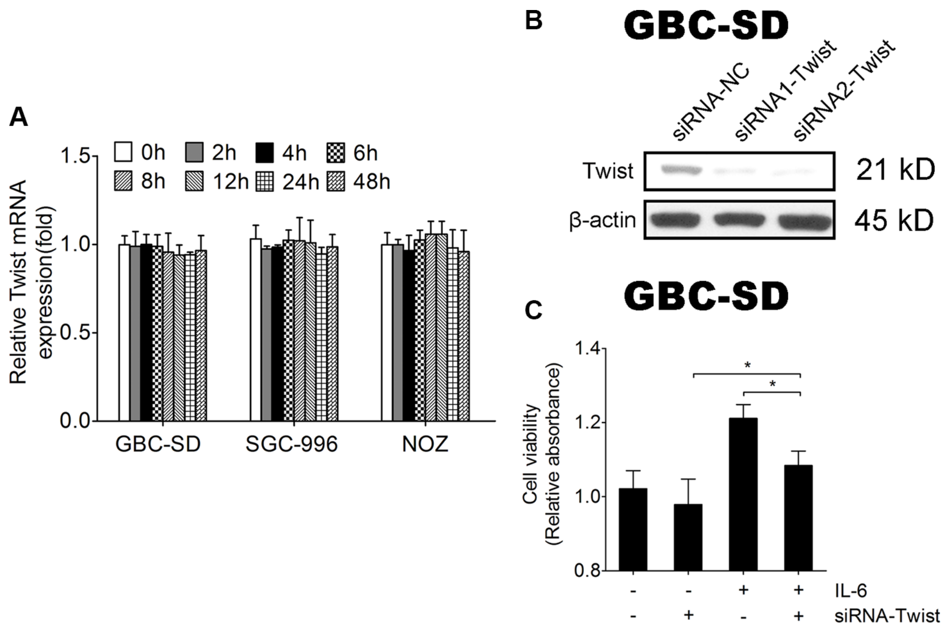

Supplementary Figure S3: (**A**) The expression of Twist mRNA in GBC cells after IL-6 treatment. The results are shown as mean ± S.D. (**B**) The knockdown efficiency of siRNA-Twist was confirmed by comparison to a control siRNA (siRNA-NC). (**C**) CCK-8 assay to detect the cell viability of GBC cells after transfection of siRNA-Twist with IL-6 treatement, **P* < 0.05. NC, negative control.

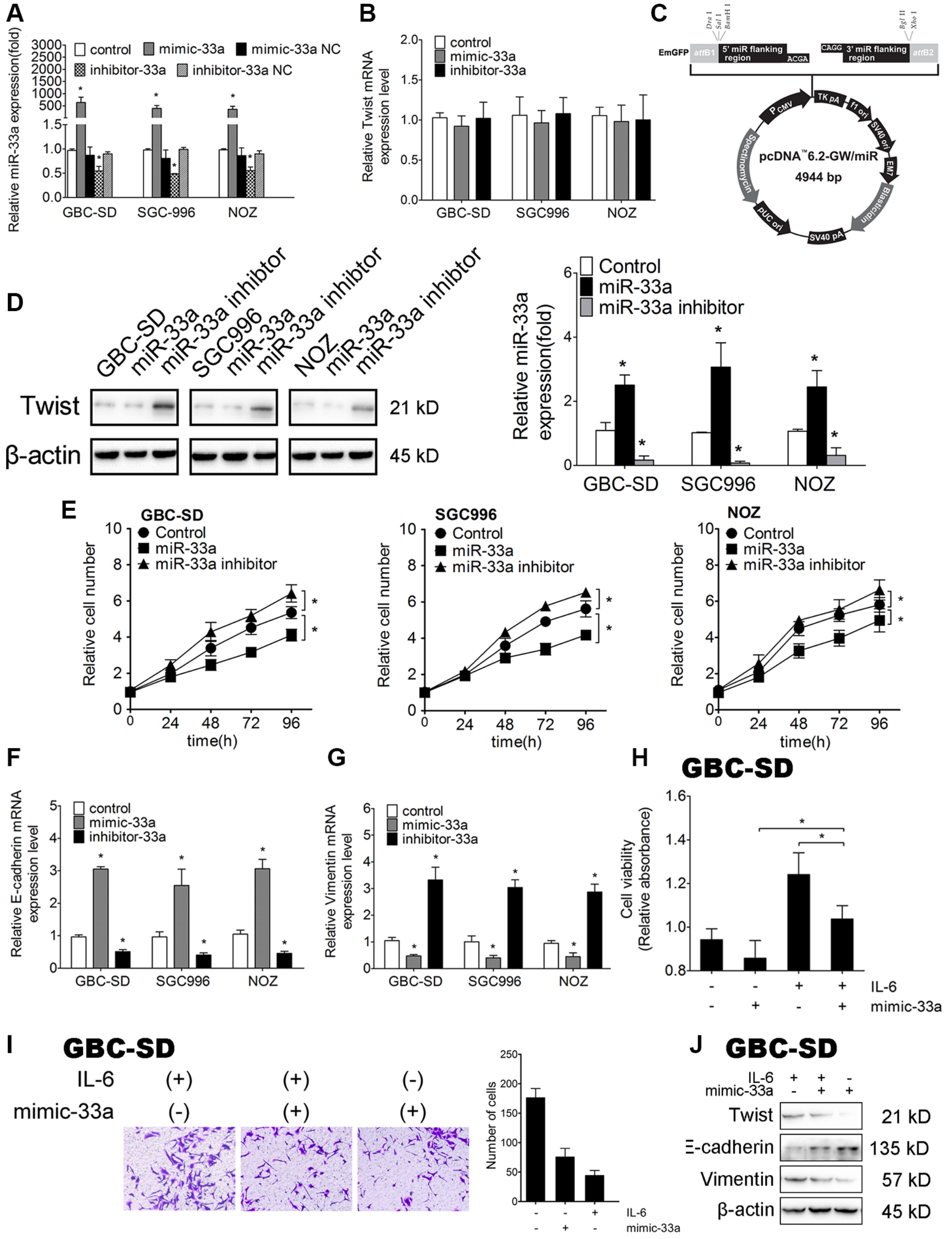

Supplementary Figure S4: MiR-33a is indispensable in IL-6-mediated GBC metastasis. (**A**) qRT-PCR analysis of miR-33a levels in GBC cells transfected with negative control, mimic-33a, or inhibitor-33a, **P* < 0.05. The results are shown as mean ± S.D. (**B**) The effect of mimic-33a, inhibitor-33a, or their negative controls on Twist mRNA levels in GBC cells. The results are shown as mean ± S.D. (**C**) A schematic diagram of pcDNA6.2-miR. (**D**) The expression of Twist protein level (left panel) and miR-33a (right panel) in GBC cells transfected with control, pcDNA6.2-miR-33a, or pcDNA6.2-miR-33a inhibitor, **P* < 0.05. The results are shown as mean ± S.D. (**E**) The effect of mimic-33a, inhibitor-33a, or their negative controls on cell viability in GBC cells (upper panel and lower two panels), **P* < 0.05. The results are shown as mean ± S.D. (**F**) The effect of mimic-33a, inhibitor-33a, or their negative controls on E-cadherin mRNA levels in GBC cells. The results are shown as mean ± S.D. (**G**) The effect of mimic-33a, inhibitor-33a, or their negative controls on Vimentin mRNA levels in GBC cells. The results are shown as mean ± S.D. (**H**) CCK-8 assay to detect cell viability in GBC-SD cells after transfection of mimic-33a with IL-6 treatment, **P* < 0.05. The results are shown as mean ± S.D. (**I**) The effect of transfection of mimic-33a with IL-6 treatment on migration in GBC-SD cells. Quantitative analysis of migrated cells (right panels), **P* < 0.05. The results are shown as mean ± S.D. (**J**) Western blotting analysis of Twist, E-cadherin and Vimentin protein levels in GBC-SD cells after transfection of mimic-33a with IL-6 treatment.

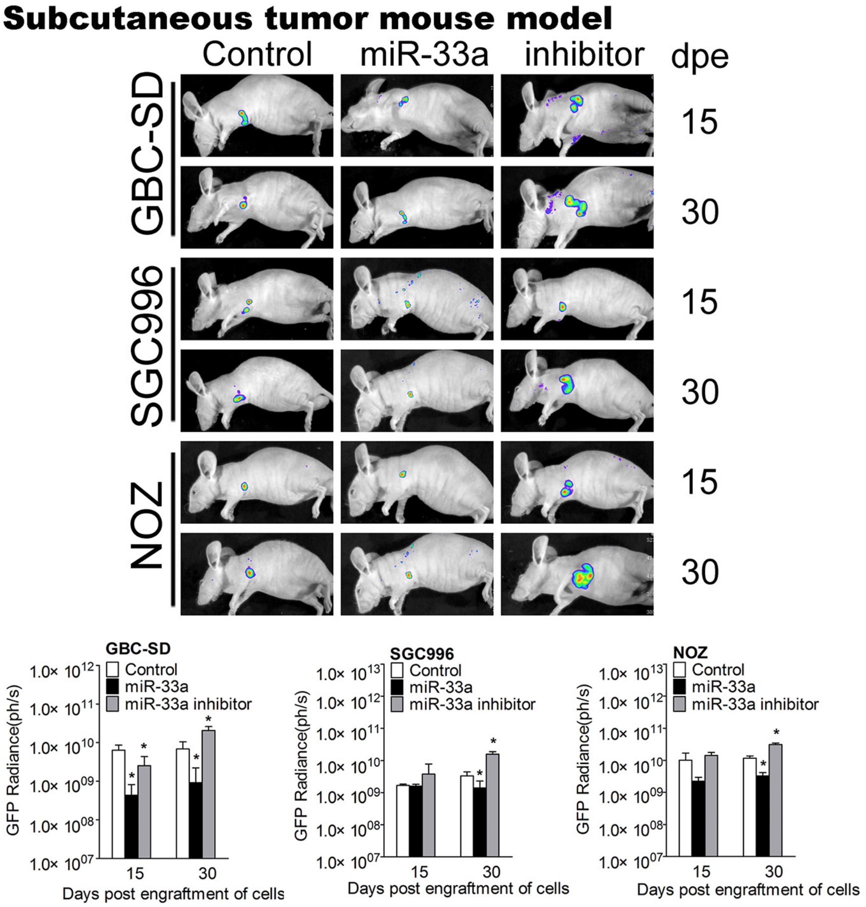

Supplementary Figure S5: The expression of GFP (lower panel) in mice subcutaneous tumor models of GBC cells transfected with control, pcDNA6.2-miR-33a, or pcDNA6.2-miR-33a inhibitor. dpe, days post engraftment (*n* = 6 for each group). The results are shown as mean ± S.D.
